# Therapeutic effects of phlorotannins in the treatment of neurodegenerative disorders

**DOI:** 10.3389/fnmol.2023.1193590

**Published:** 2023-05-18

**Authors:** Yoon Ji Kwon, Oh Ig Kwon, Hye Jeong Hwang, Hyeon-Cheol Shin, Sungchil Yang

**Affiliations:** ^1^Department of Neuroscience, City University of Hong Kong, Kowloon, Hong Kong SAR, China; ^2^Botamedi Brain Health and Medical Care Company Limited, Central, Hong Kong SAR, China; ^3^Center for Molecular Intelligence, SUNY Korea, Incheon, Republic of Korea

**Keywords:** Alzheimer’s disease, Parkinson’s disease, neurodegenerative disease, phlorotannin, polyphenol

## Abstract

Phlorotannins are natural polyphenolic compounds produced by brown marine algae and are currently found in nutritional supplements. Although they are known to cross the blood–brain barrier, their neuropharmacological actions remain unclear. Here we review the potential therapeutic benefits of phlorotannins in the treatment of neurodegenerative diseases. In mouse models of Alzheimer’s disease, ethanol intoxication and fear stress, the phlorotannin monomer phloroglucinol and the compounds eckol, dieckol and phlorofucofuroeckol A have been shown to improve cognitive function. In a mouse model of Parkinson’s disease, phloroglucinol treatment led to improved motor performance. Additional neurological benefits associated with phlorotannin intake have been demonstrated in stroke, sleep disorders, and pain response. These effects may stem from the inhibition of disease-inducing plaque synthesis and aggregation, suppression of microglial activation, modulation of pro-inflammatory signaling, reduction of glutamate-induced excitotoxicity, and scavenging of reactive oxygen species. Clinical trials of phlorotannins have not reported significant adverse effects, suggesting these compounds to be promising bioactive agents in the treatment of neurological diseases. We therefore propose a putative biophysical mechanism of phlorotannin action in addition to future directions for phlorotannin research.

## 1. Introduction

**Figure 1:**
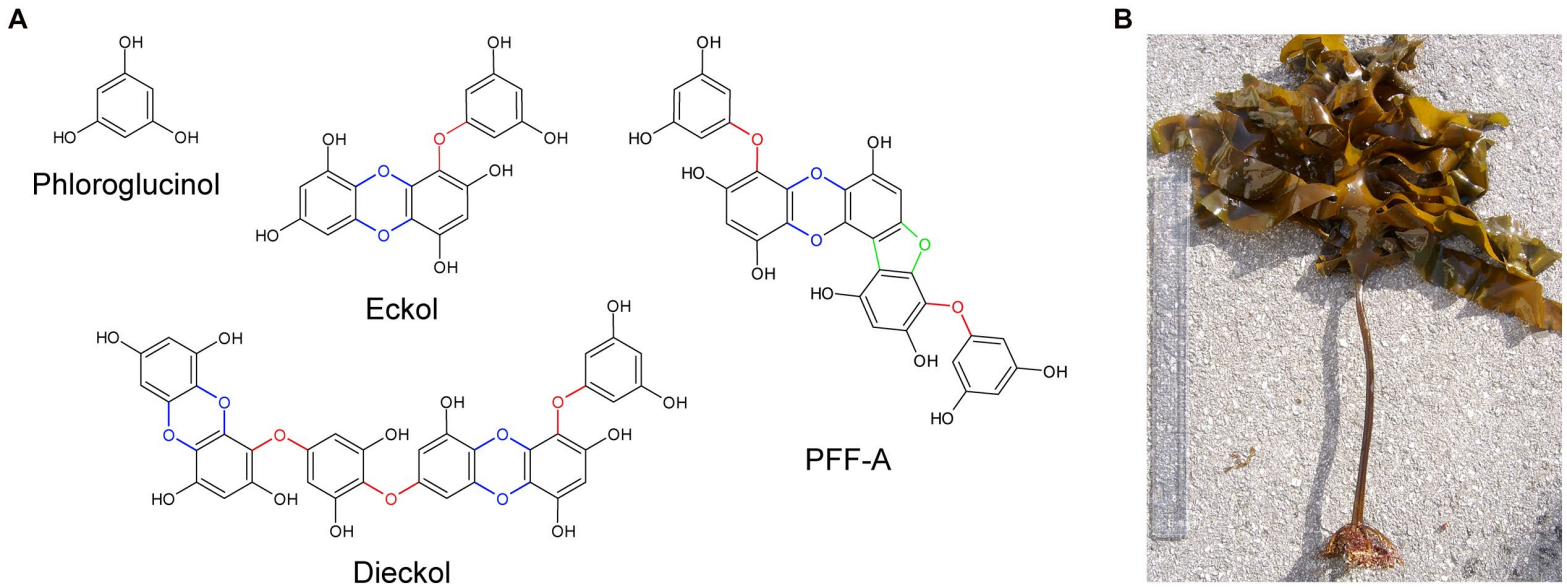
The chemical structures of phloroglucinol and phlorotannin compounds. **(A)** Phlorotannins are polyphenolic oligomers of phloroglucinol units that are connected by ether bonds (red), dibenzo-*p*-dioxin moieties (blue), and dibenzofuran elements (green). **(B)** Specimen of *Ecklonia cava*, a species of brown marine algae and common source of phlorotannins. PFF-A, phlorofucofuroeckol A.

Phlorotannins are a group of naturally occurring polyphenolic compounds found in brown marine algae. In algae, these compounds promote wound healing (Luder and Clayton, 2004; Halm et al., 2011) and contribute to defense against ultraviolet radiation (Cruces et al., 2012) and grazing (Emeline et al., 2021). They account for approximately 0.5–30% of the organism’s dry weight and are found as a heterogeneous mixture of compounds. Phlorotannins are oligomers of phloroglucinol (1,3,5-trihydroxybenzene) and contain various numbers of phenolic rings, which give rise to compounds with a wide range of molecular weights between 0.126 to 650 kDa (Ragan and Glombitza, 1986). The diversity of phlorotannins also arises from the different combinations of linkages between phloroglucinols, the addition of hydroxyl groups, and the various positions of linkages and hydroxyl groups (positional isomerism; Heffernan et al., 2015). Researchers have theorized that compared to terrestrial polyphenols, phlorotannins – in particular eckol, dieckol and phlorofucofuroeckol A (PFF-A) – have faster electron donation and higher redox potential due to the higher degree of polymerization and the presence of ether bonds, dibenzodioxin moieties, and dibenzofuran elements that link phloroglucinol units (Figure 1A); these features confer potent and self-stable antioxidant ability (Zhang et al., 2018, 2019).

Similar to polyphenols from terrestrial plants (e.g., resveratrol and quercetin), purified phlorotannins and brown algae extracts have been reported to exhibit diverse biological activities such as antioxidant, antibacterial, and anti-inflammatory activities. In addition, phlorotannins have been shown to exert potential therapeutic effects in the treatment of a wide variety of diseases, including dermatological conditions, cancers, and metabolic diseases. Notably, the phlorotannin compound dieckol has been shown to cross the blood–brain barrier (BBB; Kwak et al., 2015), and numerous *in vivo* studies using different disease models have demonstrated the potential neuroprotective effects of various phlorotannins, which are summarized in Table 1.

**Table 1:** Summary of the tested dosages and effects of purified phlorotannins and phlorotannin-rich extracts in published *in vivo* rodent studies.

Category	Substance	Min. effective dose	Timing	Route	Animal model	Stimulus	Outcome	**Reference**
Memory	Dieckol	1 mg/kg	Once daily for 7 days	Oral	ICR mice	Ethanol 3 g/kg (38% v/v solution in water) - acute alcohol intoxication	Prevention of memory loss during retention phase (passive avoidance test)	Myung et al. (2005)
Memory	PFF-A	0.2 mg/kg	Once daily for 7 days	Oral	ICR mice	Ethanol 3 g/kg (38% v/v solution in water) - acute alcohol intoxication	Prevention of memory loss during retention phase (passive avoidance test)	Myung et al. (2005)
Learning, memory	NX42 (20.1% phlorotannin)	100 mg/kg	Once daily for 4 weeks	Oral	ICR mice	Electric shocks, 15 times daily for 7 days during learning phase	Improved performance during learning and retention phases (water maze test)	Lee and Stein (2004)
AD	Phloroglucinol	1.2 μmol	Once	Bilateral stereotaxic injection into hippocampus	5XFAD mice	-	Rescued spontaneous alternation behavior in spontaneous alteration T-maze test at 12–13 days post-injection. Faster spatial learning in Morris water maze test at 7 days post-injection.	Yang et al. (2015)
AD	Phloroglucinol	100 mg/kg per day	Freely administered in drinking water for 2 months	Oral	5XFAD mice	-	Recovered spontaneous alternation ratio in spontaneous alternation T-maze and Y-maze; reductions in Aβ plaques and BACE1 protein, lipid peroxidation, Iba-1, and GFAP levels; rescued density of stubby and mushroom spines in the hippocampus CA1 subfield	Yang et al. (2018)
PD	Phloroglucinol	6 μmol	Once	Stereotaxic injection with 6-OHDA	SD rats	6-OHDA via unilateral injection	Preserved motor performance in the rotarod test; preserved number of TH+ cells in the SNpc; preserved TH and synaptophysin protein levels in the midbrain; suppressed apomorphine-induced rotation behavior	Ryu et al. (2013)
Movement	Dieckol	5 mg/kg	Once immediately before test	Oral	Swiss albino mice	-	Decreased locomotion (open field test)	Li et al. (2021)
Stroke	Polyphenol-rich extract of *Ecklonia cava* (98.5% PGE)	10 mg/kg	Half of final dose injected 30 min before and after surgery	I.p. injection	SD rats	Middle cerebral artery occlusion-induced transient focal ischemia	Reduced apoptosis at 10 mg/kg; reduced infarct size and brain water content at 50 mg/kg	Kim et al. (2012)
Seizure	Enzymatic extract of *E. cava* (98.4 mg/g PGE)	1,000 mg/kg	45 min before picrotoxin administration	Oral	SD rats	Picrotoxin (7 mg/kg)-induced clonic seizure	Increased latency to seizure onset	Cho et al. (2012)
Sleep	Dieckol	100 mg/kg	24 h after baseline recording in a 48-h study	Oral	C57BL/6 N mice	-	Reduced sleep latency and increased time spent in NREMS at 100 mg/kg; increased frequency of transitions from wake to NREMS at 150 mg/kg	Yoon et al. (2020)
Sleep	Ethanol extract of *E. cava*	250 mg/kg	12 h after baseline recording in a 24-h study	Oral	C57BL/6 N mice	-	Reduced sleep latency and increased sleep duration at 250 mg/kg; decreased time spent awake and increased time spent in NREMS at 500 mg/kg	Yoon et al. (2014)
Sleep	Ethanol extract of *E. cava* Kjellman (138.5 mg/g PGE)	1,000 mg/kg	45 min before pentobarbital administration	Oral	ICR mice	Pentobarbital (30 mg/kg [sub-hypnotic dose] and 45 mg/kg [hypnotic dose])-induced sedation	Reduced sleep latency and increased sleep duration	Cho et al. (2012)
Sleep	Polyphenol-rich extract of *E. cava* (630.2 mg/g PGE)	50 mg/kg	45 min before pentobarbital administration	Oral	SD rats	Pentobarbital (30 mg/kg [sub-hypnotic dose] and 45 mg/kg [hypnotic dose])-induced sedation	Reduced sleep latency and increased sleep duration	Cho et al. (2012)
Pain	Dieckol	5 mg/kg	30–60 min before pain stimulus	Oral	Swiss albino mice	-	Increased reaction latency to hot plate at 5 mg/kg (when supplemented with morphine); increased reaction latency to tail immersion at 10 mg/kg	Li et al. (2021)

The minimum effective dose indicates the lowest dosage (mg/kg) of the tested substance that elicited a significant change in a parameter (*p* < 0.05). mg/g PGE indicates the total polyphenol content in phloroglucinol equivalents. AD, Alzheimer’s disease; PD, Parkinson’s disease; Aβ, amyloid beta; PFF-A, phlorofucofuroeckol A; PGE, phloroglucinol equivalents; i.p., intraperitoneal; NREMS, non-rapid eye movement sleep; SD, Sprague Dawley; GFAP, glial fibrillary acidic protein; SNpc, substantia nigra pars compacta; TH, tyrosine hydroxylase.

This review discusses current evidence on the potential therapeutic benefits of phlorotannins in the treatment of neurodegenerative disorders, with a particular focus on Alzheimer’s disease (AD) and Parkinson’s disease (PD). Herein, we only include literature on phloroglucinol and the most extensively studied phlorotannins, namely eckol, dieckol, and PFF-A, as well as the ethanol and butanol extracts of *Ecklonia* species of brown algae (Figure 1B), which is a common source of phlorotannins. We then briefly discuss the most recent findings related to the effects of phlorotannins in the context of stroke, sleep disorders and nociception, and propose future directions for research.

## 2. Cognitive effects of phlorotannins

The finding that dieckol can cross the BBB in rats after administration via intravenous injection has provided a strong rationale for the use of phlorotannins in the treatment of central nervous system (CNS) diseases (Kwak et al., 2015). In that study, fluorescein isothiocyanate (FITC)- and rhodamine B (RhoB)-labeled dieckol were detected in the cortex and hippocampus in as early as 20 min after injection. No phlorotannin membrane transporters have been identified, and it is possible that the lipid solubility of dieckol permits its entry across the BBB by membrane diffusion. In computational models, eckol, dieckol, and PFF-A form multiple hydrophobic interactions (Paudel et al., 2019; Aatif et al., 2021) that may facilitate their transcellular transport across the BBB despite their relatively high molecular weights (372.3, 742.5, and 602.5 Da, respectively).

Numerous studies on phlorotannins have demonstrated their effects on cognitive processes such as learning and memory (Table 1), which are accompanied by changes in neurotransmitter levels such as acetylcholine (ACh), glutamate, γ-aminobutyric acid (GABA), 5-hydroxytryptamine (5-HT), and norepinephrine (NE; Myung et al., 2005). In one study, mice exposed to fear-stressed learning were subjected to the Morris water maze test (Lee and Stein, 2004). Mice that consumed brown kelp extract daily for 4 weeks performed significantly better than control mice during the learning and retention phases, suggesting that phlorotannins enhance cognitive resistance to stress. The effect of these compounds on memory performance was further tested in a mouse model of acute ethanol (EtOH) intoxication by using a passive-avoidance test (Myung et al., 2005). Oral pretreatment with PFF-A or dieckol once daily for 7 days before intoxication mitigated the loss of learned behavior in intoxicated mice; increasing the dose of PFF-A and dieckol produced better memory performance. The effects of these phlorotannins were comparable to those of tacrine, a cholinesterase-inhibiting drug approved for the symptomatic treatment of AD. High-performance liquid chromatography revealed significant alterations in the endogenous levels of ACh, GABA, 5-HT, and NE in mice treated with phlorotannins (Myung et al., 2005). These results suggest that phlorotannins are biologically active in the brains of animals, where they influence neurotransmitter levels, cognitive functioning, and behavior.

## 3. Effects of phlorotannins against Alzheimer’s disease

AD is the most common cause of cognitive impairment and dementia in humans. Its symptoms begin with mild cognitive impairment such as short-term memory loss, difficulty learning new information, and spatiotemporal disorientation (Gauthier et al., 2022), which progress into dementia over time. The biological hallmarks of AD are (1) the loss of ACh and cholinergic innervation, (2) the formation of extracellular amyloid beta (Aβ) plaques, and (3) chronic inflammation due to excessive microglial activation in the brain. Currently, the pathogenesis of AD is thought to involve the direct interference of cholinergic signaling by Aβ species via interaction with ACh receptors (George et al., 2021). Aβ also triggers tau protein hyperphosphorylation, converting it into a toxic state (Zheng et al., 2002; Lloret et al., 2011). This combination of Aβ and hyperphosphorylated tau further reduces the synthesis of ACh (Pedersen et al., 1996), decreases the number of dendritic spines (Chabrier et al., 2014), silences neuronal activity (Busche et al., 2019), and inhibits synaptic plasticity (Ondrejcak et al., 2019). With disease progression, hyperphosphorylated tau causes axonal degeneration and neuronal death (Bloom, 2014; Pereira et al., 2021), resulting in severe cognitive impairment. To date, only one disease-modifying drug, aducanumab – an anti-amyloid monoclonal antibody that lowers Aβ plaque levels in the brain – has been approved for AD treatment.

### 3.1. Acetylcholinesterase inhibition by phlorotannins

In the synaptic cleft, ACh is degraded by acetylcholinesterase (AChE) or butyrylcholinesterase (BuChE). To counteract the loss of ACh and cholinergic synapses in AD, reversible AChE and BuChE inhibitors (e.g., rivastigmine and donepezil) are often used to treat the symptoms of AD dementia. Several studies have revealed the inhibitory effects of phlorotannins on AChE and BuChE *in vitro*. Purified PFF-A, eckol, and dieckol were shown to inhibit AChE (Myung et al., 2005; Yoon et al., 2008; Kannan et al., 2013; Choi et al., 2015) and BuChE activity (Choi et al., 2015; Nho et al., 2020) in colorimetric enzyme assays. Phlorotannin-rich fractions of brown kelp extract were also shown to inhibit AChE and BuChE activity (Lee and Stein, 2004; Choi et al., 2015). It has not yet been determined whether phlorotannins affect ACh levels *in vivo* using animal models of AD. However, in a mouse model of EtOH intoxication, dieckol and PFF-A treatment prevented the decrease of ACh levels in the hippocampus, cortex and striatum, and alleviated memory loss (Myung et al., 2005).

### 3.2. Inhibitory effect of phlorotannins on amyloid beta biosynthesis, aggregation and neurotoxicity

Aβ is produced by the proteolysis of amyloid precursor protein (APP). APP can be cleaved by α-secretase to yield soluble APP alpha (sAPPα) peptides, or by β-secretase (BACE1) to produce soluble APP beta (sAPPβ) peptides. Both enzymatic reactions also produce transmembrane protein fractions – C-terminal fragment (CTF) α and CTFβ, respectively – which are subsequently cleaved by the γ-secretase complex. Amyloidogenic Aβ fragments are produced by the proteolysis of CTFβ by γ-secretase. In the CNS, sAPPα, sAPPβ and Aβ are essential for synaptic transmission, plasticity, cytoprotection, and neuronal glucose homeostasis maintenance (Giuffrida et al., 2015; Rice et al., 2019). However, Aβ overproduction and insufficient Aβ clearance result in the abnormal aggregation of Aβ in the extracellular matrix (Bateman et al., 2006). Furthermore, an imbalance between sAPPα and sAPPβ levels reduces neuron viability (Barger and Harmon, 1997). Higher BACE1 activity is found in brain extracts of AD patients (Hampel et al., 2021). In some cases of familial AD, the Swedish gene mutation (*APP*^Swe/Swe^) increases the likelihood of APP cleavage by BACE1, culminating in increased Aβ production (Haass et al., 1995). Preclinical studies have demonstrated that BACE1 is a promising therapeutic target in patients with early-stage AD. The sequential deletion of BACE1 was shown to reverse amyloid deposition and improve contextual memory in adult transgenic mice harboring multiple mutations associated with human familial AD (i.e., 5XFAD mice, a mouse model of Aβ-induced neurodegeneration and Aβ plaque formation; Hu et al., 2018).

**Figure 2:**
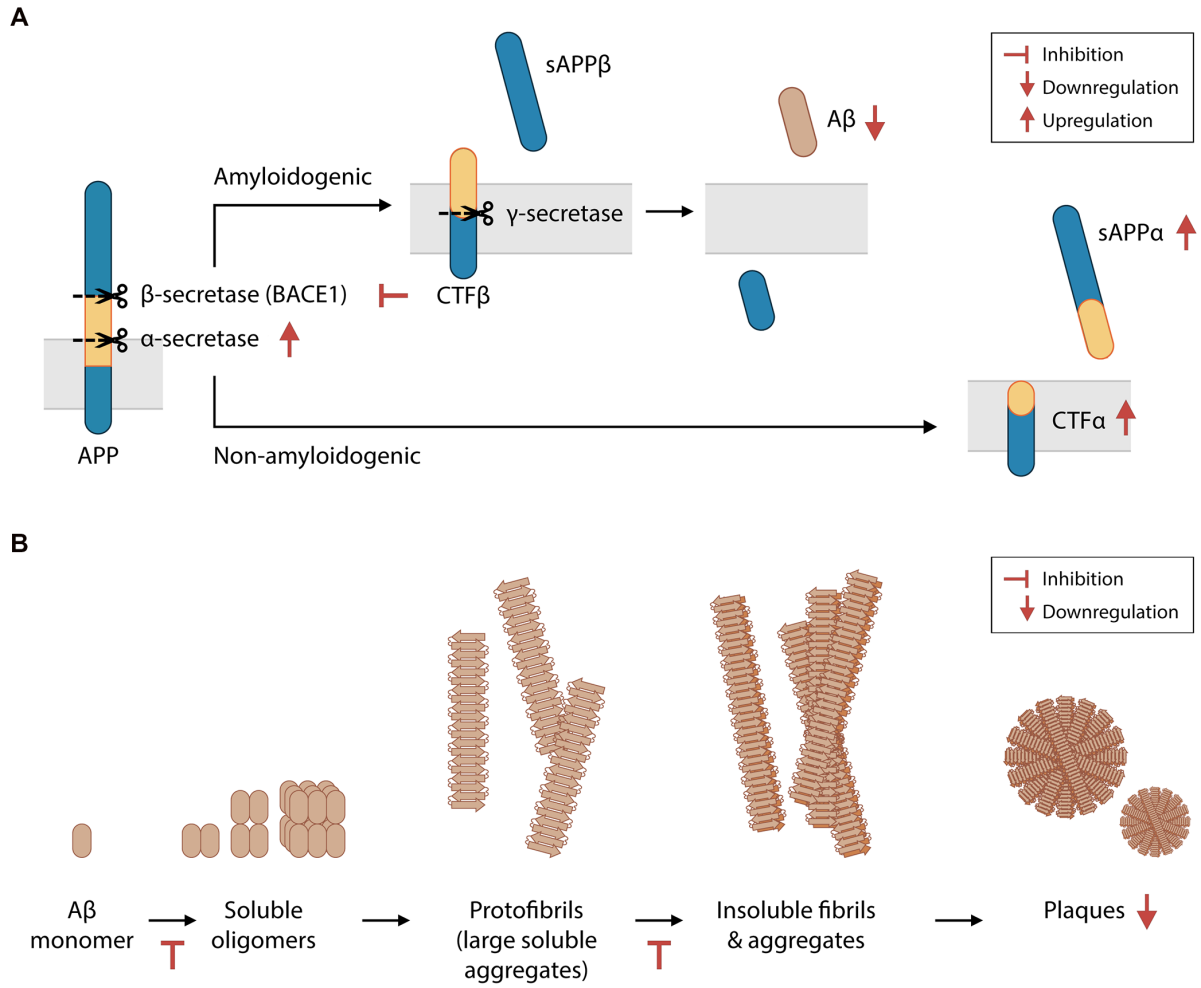
Phlorotannins inhibit the biosynthesis and oligomerization of amyloid beta fragments. **(A)** Aβ is produced via the proteolytic cleavage of APP protein by β-secretase and then γ-secretase. Phlorotannins inhibit β-secretase (BACE1) and γ-secretase activity and promote APP processing by α-secretase, suppressing Aβ production. **(B)** Aβ monomers aggregate into soluble oligomers, fibrils, and plaques. Phlorotannins inhibit the oligomerization into soluble oligomers as well as fibrilization into fibrils. Ultimately, plaque formation is reduced. Red flat-ended arrows indicate inhibition, red upward arrows indicate upregulation, and red downward arrows indicate downregulation. Aβ, amyloid beta; APP, amyloid precursor protein; BACE1, beta secretase 1; sAPPα, soluble amyloid protein precursor alpha; sAPPβ, soluble amyloid protein precursor beta; CTFα, C-terminal fragment alpha; CTFβ, C-terminal fragment beta. Adapted from Kang et al. (2011) and Yang et al. (2018).

In 4-month-old 5XFAD mice, animals that were orally administered phloroglucinol for 2 months had 33% fewer Aβ plaques in the hippocampus than saline-treated control mice (Yang et al., 2018; Figure 2A). 5XFAD mice were found to have increased BACE1 levels in the brain, which were also suppressed by phloroglucinol. Treatment with a phlorotannin-rich butanol extract of *E. cava* (BEC) led to similar suppression of BACE1 protein and mRNA levels in a HEK293 human embryonic kidney cell line engineered to stably express the APP^Swe^ protein (Kang et al., 2013). This cell line also secreted significantly lower amounts of Aβ_1-40_ and Aβ_1-42_ peptides into the supernatant after BEC treatment. In other studies, purified eckol, dieckol, PFF-A, and other phlorotannins were shown to non-competitively inhibit BACE1 activity in cell-free assays (Jung et al., 2010; Choi et al., 2015). Therefore, phlorotannins may inhibit Aβ biosynthesis directly by inhibiting BACE1 and/or indirectly by regulating BACE1 protein synthesis or degradation. The crude cell extract after BEC treatment was also shown to downregulate *PSEN1* transcription (a subunit of the γ-secretase complex) and γ-secretase activity, while upregulating the protein levels of sAPPα and CTFα and the activity of α-secretase (Kang et al., 2011, 2013).

Phlorotannins not only interfere with the biosynthesis of Aβ but also inhibit its oligomerization, fibrillization, and neurotoxicity (Figure 2B). In the extracellular matrix, Aβ monomers spontaneously aggregate to form soluble oligomers, protofibrils (large soluble aggregates), insoluble fibrils, and eventually plaques. Phloroglucinol, eckol, dieckol and PFF-A were shown to directly inhibit Aβ_25–35_ aggregation in a cell-free assay, and the binding of dieckol and PFF-A to Aβ were predicted to induce structural changes of Aβ *in silico* (Seong et al., 2019). When BEC was incubated with Aβ_1-42_ peptides in a cell-free solution, the concentrations of large soluble oligomers and Aβ fibrils decreased in a dose-dependent manner (Kang et al., 2011). Polyphenols may disrupt Aβ aggregation via metal chelation (Lakey-Beitia et al., 2021), and phlorotannins may be candidate compounds for chelation therapy in patients with AD (Bolognin et al., 2009; Lakey-Beitia et al., 2021). The potential inhibition of Aβ aggregation *in vivo* by phlorotannins may also contribute to the decreased number of plaques seen in 5XFAD mice treated with phloroglucinol (Yang et al., 2018).

As previously mentioned, Aβ species disrupt synaptic transmission and plasticity (Bloom, 2014; Pereira et al., 2021). The loss of synapses and dendritic spines, especially stubby and mushroom-type spines, are commonly seen in the hippocampus of AD patients (Selkoe, 2002) and 5XFAD mice (Yang et al., 2018). Aβ_1–42_ also leads to reduced spinal density on the secondary dendrites of primary rat hippocampal neurons, as indicated by the decreased expression of PSD-95 and synaptophysin (glutamatergic postsynaptic and presynaptic markers, respectively) (Yang et al., 2015). Treatment with phloroglucinol reversed this loss of spinal density in the hippocampal CA1 of 6-month-old 5XFAD mice and in cultured rat hippocampal neurons (Yang et al., 2015, 2018), without decreasing the intracellular levels of Aβ *in vitro*. However, bilateral stereotaxic injection of phloroglucinol into the dentate gyrus did not affect the levels of synaptophysin or PSD-95 in 5XFAD mice (Yang et al., 2015), although treated mice exhibited better spatial learning and memory performance than control mice while undergoing the spontaneous alternation T-maze and Morris water maze tests. The performance of 5XFAD mice on the spontaneous alternation T-maze test was also restored after 2 months of oral phloroglucinol treatment (Yang et al., 2018).

In the 5XFAD mouse model of AD, Aβ species promote cell death and neuronal loss (Jawhar et al., 2012). Specifically, they trigger multiple neurotoxic pathways such as the dysregulation of presynaptic Ca^2+^ levels (Mucke and Selkoe, 2012), excitotoxicity, oxidative stress, and caspase-3-mediated apoptosis (Eimer and Vassar, 2013). Phlorotannins may prevent Aβ-induced neuron death. Treatment with PFF-A, dieckol and eckol prevented the death of PC12 rat pheochromocytoma cells that were incubated with Aβ_25–35_ peptides (Ahn et al., 2012; Lee et al., 2018). BEC pretreatment also increased the viability of rat cortical neurons in response to Aβ oligomers (Kang et al., 2011) but did not affect the viability of neurons challenged with Aβ monomers or fibrils. These neuroprotective effects may be due to the antioxidative properties of phlorotannins (see section 4.4, Antioxidative effects of phlorotannins). Immortalized mouse hippocampal HT-22 cells that had been pretreated with phloroglucinol had significantly lower levels of intracellular reactive oxygen species (ROS) after treatment with Aβ_1-42_ compared to cells not treated with phloroglucinol (Yang et al., 2015). In addition, reduced lipid peroxidation, as indicated by 4-hydroxynonenal, was observed in the hippocampi of 5XFAD mice after oral phloroglucinol treatment for 2 months.

### 3.3. Inhibitory effects of phlorotannins on microglial activation and inflammation

Chronic neuroinflammation contributes to the progression of AD. Microglia are the resident phagocytic immune cells of the brain that form a cellular network throughout the CNS. These cells sense injury, clear dead cells and debris, mediate synaptic remodeling, and initiate neuroinflammatory responses to pathogens or abnormal proteins, including Aβ (D'Andrea et al., 2004). Microglia migrate toward Aβ plaques and fibrils (Bolmont et al., 2008) and internalize Aβ species (Perlmutter et al., 1990; Lee and Landreth, 2010) to help clear these proteins, a process that occurs mainly in the early stages of AD. Microglia are found in two states, the neuroprotective M2 phenotype or the pro-inflammatory and detrimental M1 phenotype, which differ in morphology, function, and biomarker expression. In the AD brain, activated M1 microglia are typically clustered around plaques (McGeer et al., 1987). Aβ species activate microglia to the M1 phenotype (Bamberger et al., 2003; Koenigsknecht-Talboo and Landreth, 2005), promoting the biosynthesis and release of ROS, nitric oxide (NO), and pro-inflammatory cytokines. This process ultimately leads to neuronal dysfunction, apoptosis, axonal injury, and synaptic loss (Leng and Edison, 2021). In patients, a rapid increase in the M1 microglial population is associated with accelerated AD progression and decline of cognitive function (Hamelin et al., 2018; Bradburn et al., 2019).

**Figure 3:**
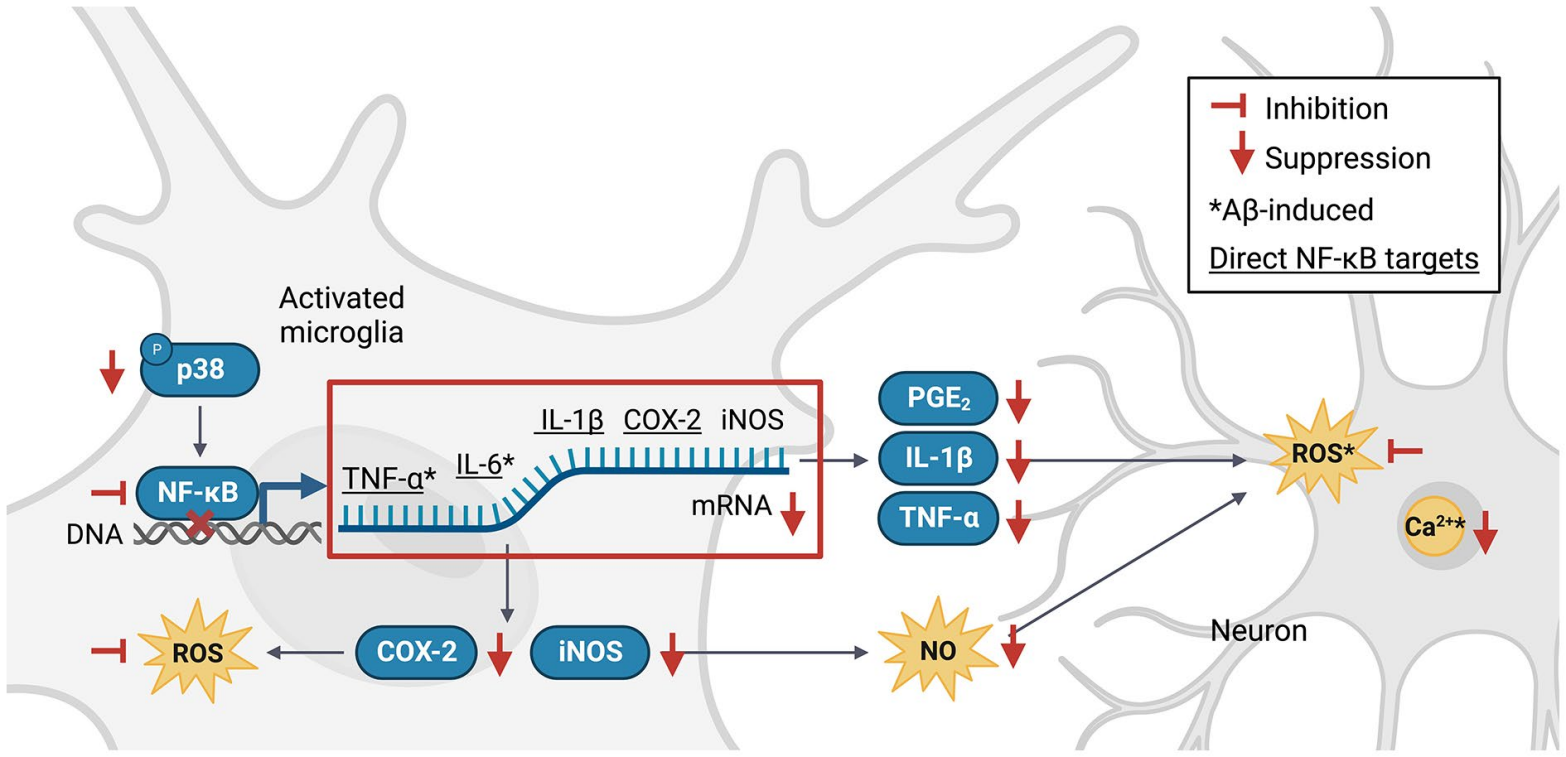
Phlorotannins inhibit the NF-κB pathway of microglial activation and the pro-inflammatory signaling cascade. Phosphorylated p38 activates NF-κB, which in turn promotes transcription of pro-inflammatory signaling molecules. Neuroinflammation generates ROS, NO and Ca^2+^, induces oxidative damage, and promotes neuron death. Phlorotannins inhibit NF-κB activity and also directly scavenge ROS, thereby reducing microglial recruitment and neuroinflammation, ultimately increasing neuron viability. Red flat-ended arrows indicate direct inhibition; red downward arrows indicate downregulation; asterisks indicate changes observed in Aβ and phlorotannin-treated cells; underlined proteins indicate direct downstream targets of NF-κB. NF-κB, nuclear factor kappa-light-chain-enhancer of activated B cells; TNF-α, tumor necrosis factor alpha; IL-6, interleukin 6; IL-1β, interleukin 1 beta; PGE_2_, prostaglandin E2; COX-2, cyclooxygenase-2; iNOS, inducible nitric oxide synthase; NO, nitric acid; ROS, reactive oxygen species; Aβ, amyloid beta. Adapted from previous studies in Table 2.

The nuclear factor kappa-light-chain-enhancer of activated B cells (NF-κB) pathway is an important signaling pathway involved in the activation of M1 microglia (Wang et al., 2018). Activated NF-κB translocates into the nucleus, where it promotes the expression of genes encoding key pro-inflammatory cytokines and enzymes such as tumor necrosis factor-alpha (TNF-α), interleukin 1 beta (IL-1β), IL-6 and cyclooxygenase-2 (COX-2; Liu et al., 2017), which in turn upregulate the expression of secondary messengers such as prostaglandin E2 (PGE_2_), inducible NO synthase (iNOS), NO, and ROS (Combs et al., 2001). NF-κB can be activated by TNF-α, ROS or Ca^2+^ (Snow and Albensi, 2016) in a positive feedback loop, or via the p38 mitogen-activated protein kinase (MAPK) pathway (Li et al., 2021). Phlorotannins may inhibit pro-inflammatory signaling and microglial activation (Figure 3). One study revealed that compared to untreated control mice, 5XFAD mice treated with phloroglucinol had significantly lower protein expression of Iba-1, a microglia-specific marker, in the dentate gyrus and CA1 subfields of the hippocampus (Yang et al., 2018). The treated mice also had lower TNF-α and IL-6 mRNA levels in the hippocampus. The expression of glial fibrillary acidic protein (GFAP), a marker of mature astrocytes, was also attenuated, suggesting an overall suppressive effect of phloroglucinol on the neuroinflammatory response to Aβ plaques in these mice (Verkhratsky et al., 2010; Yang et al., 2021).

No animal studies have explored Aβ-induced microglial activation after phlorotannin treatment. However, many studies have reported the anti-inflammatory effects of phlorotannins on lipopolysaccharide (LPS, a bacterial toxin)-induced activated microglia (Table 2). Phlorotannins have been shown to suppress microglial activation by downregulating p38 phosphorylation and inhibiting the promoter activity of NF-κB. Pro-inflammatory effectors downstream of NF-κB, such as IL-1β, PGE_2_, and COX-2, are also suppressed in a phlorotannin dose-dependent manner, ultimately reducing ROS and NO generation. Notably, treatment with dieckol, eckol, and PFF-A suppressed ROS and Ca^2+^ generation in PC12 cells after Aβ challenge (Ahn et al., 2012; Figure 3). The suppression of ROS generation may be partly due to the direct antioxidative effects of phlorotannins (see section 4.4, Antioxidative effects of phlorotannins).

**Table 2:** Summary of literature on the effects of phlorotannins on lipopolysaccharide-stimulated mouse microglia *in vitro*.

Outcome	Substance	Model
Inhibited p38, ERK1/2, and JNK phosphorylation	Dieckol pretreatment (Jung et al., 2009) PFF-A pretreatment (Kim et al., 2012) EEC (Jung et al., 2009)	BV-2 microglia
Reduced NF-κB promoter activity and DNA binding	PFF-A pretreatment (Kim et al., 2012) EEC (Jung et al., 2009)	BV-2 microglia
Decreased TNF-α transcription and extracellular release	Dieckol pretreatment (Jung et al., 2009) PFF-A pretreatment (Kim et al., 2012) EEC (Jung et al., 2009) Dieckol, FITC-labeled dieckol, RhoB-dieckol (Kwak et al., 2015)	BV-2 microglia
Decreased IL-1β transcription and extracellular release	Dieckol pretreatment (Jung et al., 2009) PFF-A pretreatment (Kim et al., 2012) EEC (Jung et al., 2009)	BV-2 microglia
Decreased PGE_2_ release	Dieckol pretreatment (Jung et al., 2009) EEC (Jung et al., 2009) Dieckol, FITC-labeled dieckol, RhoB-dieckol (Kwak et al., 2015)	BV-2 microglia
Decreased COX-2 transcription and intracellular protein expression	Dieckol pretreatment (Jung et al., 2009) PFF-A pretreatment (Kim et al., 2012) EEC (Jung et al., 2009)	BV-2 microglia, Primary cortical microglia
Decreased iNOS transcription and intracellular protein expression	Dieckol pretreatment (Jung et al., 2009) PFF-A pretreatment (Kim et al., 2012) EEC (Jung et al., 2009)	BV-2 microglia, primary cortical microglia
Suppressed NO release	Dieckol (Cui et al., 2015) Dieckol pretreatment (Jung et al., 2009) PFF-A pretreatment (Kim et al., 2012) EEC (Jung et al., 2009) Dieckol, FITC-labeled dieckol, RhoB-dieckol (Kwak et al., 2015)	Primary cortical microglia, BV-2 microglia, primary cortical neurons
Suppressed intracellular ROS generation	Dieckol (Cui et al., 2015) Dieckol pretreatment (Jung et al., 2009) PFF-A pretreatment (Kim et al., 2012) EEC (Jung et al., 2009)	BV-2 microglia

Phlorotannins inhibit activation of the NF-κB pathway and downregulate the pro-inflammatory signals downstream of this pathway. ERK, extracellular signal-regulated protein kinase; JNK, c-Jun N-terminal kinase; PFF-A, phlorofucofuroeckol A; EEC, EtOH extract of *Ecklonia cava*; NF-κB, nuclear factor kappa-light-chain-enhancer of activated B cells; TNF-α, tumor necrosis factor alpha; FITC, fluorescein isothiocyanate; IL-1β, interleukin 1 beta; PGE_2_, prostaglandin E2; COX-2, cyclooxygenase-2; iNOS, inducible nitric oxide synthase; NO, nitric acid; ROS, reactive oxygen species.

## 4. Effects of phlorotannins against Parkinson’s disease

PD is the second most common neurodegenerative disease of the CNS after AD. It is characterized by the loss of dopaminergic neurons and the presence of α-synuclein deposits in the substantia nigra pars compacta (SNpc), a region of the midbrain that controls motor behavior. In patients with PD, dopamine (DA) deficiency affects the motor circuit regulated by the basal ganglia and is manifested as the core motor symptoms of PD – slowness of movement, rigidity and resting tremor, which are collectively known as parkinsonism (Postuma et al., 2015). Patients may also present with non-motor symptoms such as depression, cognitive impairment and sleep disorders, which often worsen with disease progression (Armstrong and Okun, 2020). Currently, no neuroprotective or disease-modifying drug has been approved for the treatment of PD.

To date, only one study has explored the effects of phlorotannins in an animal model of PD, which used stereotaxic injection of 6-hydroxydopamine (6-OHDA) to cause the death of nigral dopaminergic neurons. Simultaneous injection of 6-OHDA and phloroglucinol into the right medial forebrain bundle of Sprague–Dawley (SD) rats resulted in a significant preservation of motor performance on the rotarod test, compared with 6-OHDA-injected control rats (Ryu et al., 2013). The study also reported a higher percentage of tyrosine hydroxylase-positive cells on the ipsilateral (6-OHDA-affected) side of the medial forebrain bundle than on the contralateral side, suggesting that phloroglucinol protected DA neurons against 6-OHDA-induced death. The decrease in synaptophysin levels in the midbrain was also mitigated by phloroglucinol, indicating that this compound may help to preserve glutamatergic synapses in the midbrain. In addition, lower apomorphine-induced rotation behavior was observed in the phloroglucinol group than in the control group.

### 4.1. Inhibitory effects of phlorotannins on monoamine oxidases

Dopamine replacement therapy involves the prescription of levodopa (L-DOPA) or other dopamine receptor agonists and is the gold standard treatment for symptoms of parkinsonism. However, patients with parkinsonism require higher and more frequent doses of dopamine analogues over time due to the progressive pathological changes associated with the disease and/or its effects on homeostatic DA regulation in the brain (Armstrong and Okun, 2020). Monoamine oxidases (MAOs) metabolize and inactivate monoamine neurotransmitters (i.e., DA, NE, serotonin) in the brain, and thus monoamine oxidase inhibitors (MAOIs) – particularly reversible inhibitors – are frequently prescribed as an adjunct to L-DOPA treatment and can effectively treat both motor and non-motor symptoms of PD (Stocchi et al., 2015; Cereda et al., 2017).

Eckol, dieckol, PFF-A and phloroglucinol were shown to inhibit the enzymatic activity of both MAO isoforms, MAO-A and MAO-B, in a cell-free colorimetric assay (Jung et al., 2017; Seong et al., 2019). Dieckol and eckol non-competitively inhibited MAO-B (IC_50_ = 43.42 μM and 83.44 μM, respectively). Dieckol also non-competitively inhibited MAO-A (IC_50_ = 11.43 μM), while eckol reversibly inhibited MAO-A (IC_50_ = 7.20 μM). Computationally, the phlorotannin compounds were docked to the active sites of both isoforms. These results suggest that phlorotannins can serve as orthosteric modulators of MAOs and thus may be used to improve motor skills and non-cognitive functions.

### 4.2. Inhibitory effects of phlorotannins on mitochondrial dysfunction and glutamate-induced excitotoxicity

One possible cause of the loss of DA neurons in the SNpc is glutamate excitotoxicity, which can occur via overactivation of the N-methyl-D-aspartate receptor (NMDAR), and consequently, Ca^2+^ loading into the mitochondria (Blythe et al., 2009; Verma et al., 2022). Excessive Ca^2+^ induces metabolic stress (Surmeier, 2007) and transient (i.e., “flickering”) depolarization of the inner mitochondrial membrane (ΔΨm) in SNpc neurons (Guzman et al., 2010). Mitochondrial dysfunction disrupts oxidative respiration and ATP production, resulting in increased production of free radicals such as the superoxide anion (O_2_^−^) and H_2_O_2_, and additional oxidative damage. Several studies report that defects in the mitochondrial electron transport chain complex I are involved in PD pathogenesis, and it appears to be an early and critical event in the degeneration of SNpc DA neurons (Mizuno et al., 1990; Ekstrand et al., 2007; Gonzalez-Rodriguez et al., 2021).

The effects of dieckol and PFF-A on glutamate-induced toxicity have been studied in primary rat cortical neurons (Cui et al., 2019), HT-22 cells (Kang et al., 2012; Cui et al., 2019), and PC12 cells (Kim et al., 2016). In all three cell lines, pretreatment with dieckol or PFF-A led to increased viability and significantly reduced mitochondrial membrane depolarization after glutamate challenge. Dieckol pretreatment resulted in an increase in intracellular ATP levels and a decrease in the intracellular ROS, mitochondrial ROS, and Ca^2+^ levels in a dose-dependent manner (Cui et al., 2019). In PC-12 cells, PFF-A pretreatment suppressed spikes in the intracellular O_2_^−^ and H_2_O_2_ levels and prevented the loss of mitochondrial mass (Kim et al., 2016). In the absence of glutamate toxicity, dieckol and PFF-A pretreatment did not affect ATP, ROS, or Ca^2+^ levels. These results suggest that dieckol and PFF-A protect against glutamate-induced excitotoxicity.

### 4.3. Inhibitory effects of phlorotannins on GSK-3**β**

Another hallmark of PD is the presence of LBs – abnormal protein aggregates that contain α-synuclein fibrils – in the SNpc and hippocampus. LB formation involves the recruitment, internalization, and phosphorylation of α-synuclein, which is elongated into fibrils that aggregate laterally; finally, the aggregates are packaged into LBs. This process itself also induces mitochondrial dysfunction, oxidative stress, synaptic dysfunction, and neurodegeneration (Mosharov et al., 2009). Although neurofibrillary tangles containing hyperphosphorylated tau proteins are more commonly associated with AD, they are also associated with parkinsonism in older adults with PD (Schneider et al., 2006). Hyperphosphorylation is a core pathological process of both synucleinopathy and tauopathy, and both hyperphosphorylated α-synuclein and tau are found in the PD brain (Muntane et al., 2008; Wills et al., 2010).

Glycogen synthase kinase-3 beta (GSK-3β) is a serine–threonine kinase that phosphorylates many sites on tau and α-synuclein (Golpich et al., 2015). The expression of GSK-3β is increased in brain regions associated with PD pathology, and this protein co-localizes with α-synuclein in LBs (Nagao and Hayashi, 2009; Wills et al., 2010). GSK-3β may also be involved in neuronal apoptosis and mitochondrial activity (Yang et al., 2017). Inhibition of GSK-3β was shown to protect dopaminergic neurons in various neurotoxic models of PD (Petit-Paitel et al., 2009; Li et al., 2014) and is a target of ongoing drug development efforts (Przewodowska et al., 2021). An analysis of crude extracts of Jurkat human T-cell leukemia clone E-6 cells revealed that incubation with PFF-A (and dieckol and eckol to lesser extent) inhibited GSK-3β activity (Choi et al., 2015). Furthermore, dieckol suppressed rotenone-induced α-synuclein aggregation in SH-SY5Y catecholaminergic human neuroblastoma cells (Cha et al., 2016), a process that is mediated by GSK-3β and Ca^2+^ (Yuan et al., 2015). Therefore, phlorotannins may counteract synucleinopathy and tauopathy by suppressing GSK-3β.

### 4.4. Antioxidative effects of phlorotannins

**Figure 4:**
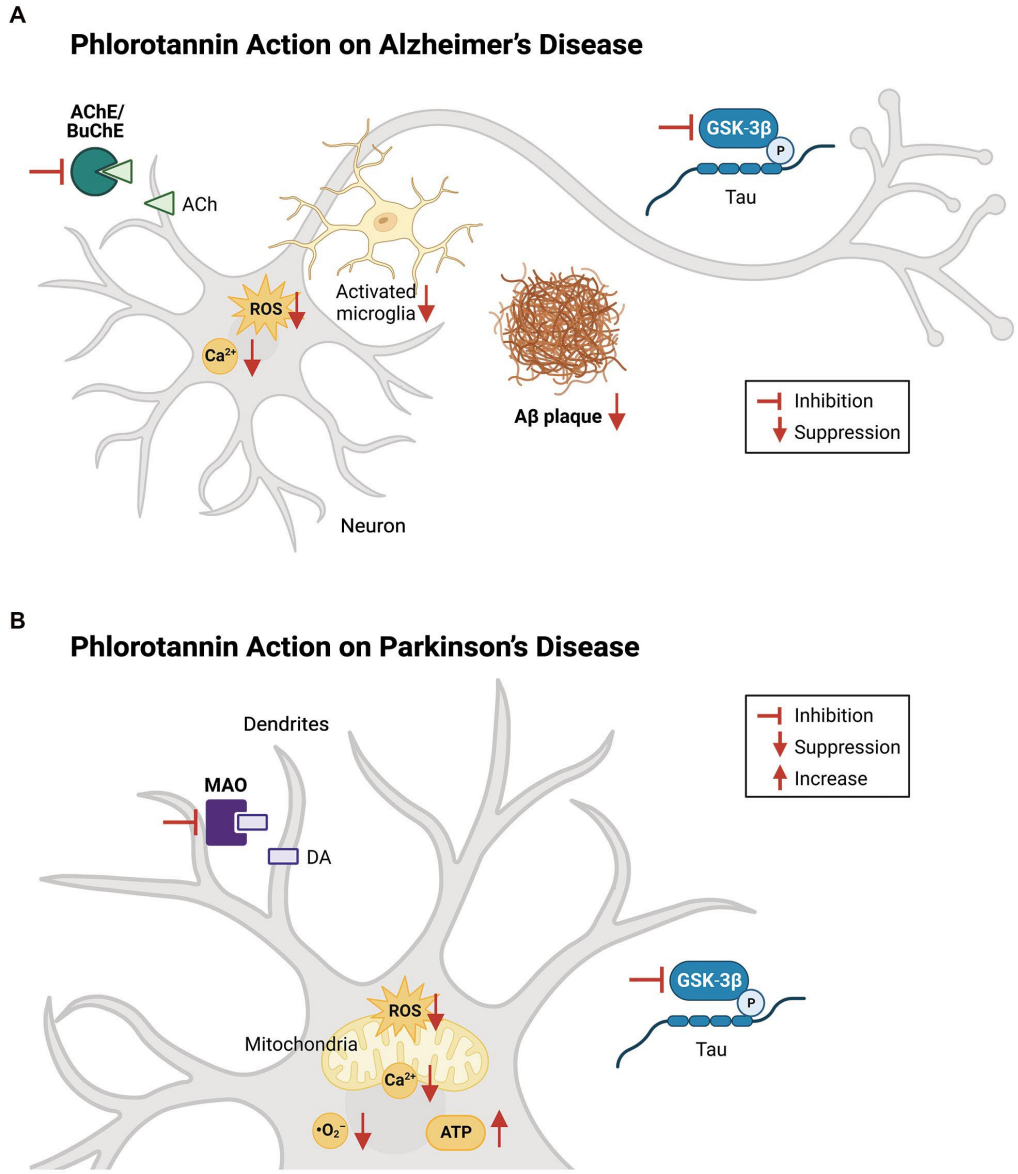
Effects of phlorotannins in Alzheimer’s and Parkinson’s disease. **(A)** In AD, phlorotannins inhibit AChE and BuChE and combat the loss of cholinergic transmission. Phlorotannins also reduce the formation of Aβ plaques. Aβ-induced spikes in intracellular ROS and Ca^2+^ levels, as well as microglial activation, recruitment, and pro-inflammatory signaling, are suppressed. Adapted from Lee and Stein (2004) and Choi et al. (2015). **(B)** In PD, phlorotannins may inhibit MAOs to counteract the loss of dopamine. In mitochondria, phlorotannins reduce mitochondrial loss, glutamate-induced membrane flickering, ROS levels, and Ca^2+^. Phlorotannins also reduce cytoplasmic ROS and O_2_^−^ levels, while rescuing ATP levels and the number of TH-positive cells in the substantia nigra. Flat-ended arrows indicate direct inhibition, upward arrows indicate upregulation, and downward arrows indicate downregulation. AChE, acetylcholinesterase; BuChE; butyrylcholinesterase, ACh, acetylcholine; ROS, reactive oxygen species; Aβ, beta amyloid; GSK-3β, glycogen synthase kinase-3 beta; P, phosphorylation; MAO, monoamine oxidase; DA, dopamine; ATP, adenosine triphosphate. Adapted from Choi et al. (2015), Cui et al. (2019), and Seong et al. (2019).

Oxidative stress contributes to the pathogenesis of both AD and PD (Figure 4), and the protective effects of dieckol and PFF-A are at least partly attributable to their direct ROS-scavenging activities. Studies have shown that treatment with various phlorotannin compounds and brown kelp extracts can reduce intracellular ROS levels and improve the viability of cells (Table 3). Numerous studies using cell-free assays [such as (Ahn et al., 2006; Senevirathne et al., 2006; Shibata et al., 2008)] have demonstrated the direct antioxidant effects of dieckol, eckol, and PFF-A against 2,2-diphenyl-1-picrylhydrazyl (DPPH), hydroxyl (•OH), and O_2_^−^ radicals. Studies using other tests for antioxidant capacity, such as 2,2′-azino-bis (3-ethylbenzothiazoline-6-sulfonic acid) (Kwon et al., 2013; Mwangi et al., 2013), oxygen radical absorbance capacity (Mwangi et al., 2013), total radical-trapping antioxidant parameter (Yotsu-Yamashita et al., 2013), and ferric reducing antioxidant power assays (Kang et al., 2003), have also suggested that phlorotannins directly scavenge oxygen radicals to reduce intracellular ROS levels.

**Table 3:** Summary of literature on the antioxidant effects of phlorotannins in various cell lines, tissues, and animal models.

Oxidative stress stimulant	Substance	Model	Reference
H_2_O_2_	Dieckol	Human endothelial progenitor cells (EPCs)	Lee et al. (2013)
	Dieckol, EEC	SH-SY5Y human neuroblastoma cell line	Nho et al. (2020)
	Dieckol, EEC	PC12 rat pheochromocytoma cell line	Nho et al. (2020) and Shin et al. (2021)
	Eckol	Chang liver HeLa/human cervical carcinoma cell line	Kim et al. (2014)
	Eckol	HepG2 human liver cancer cell line	Jun et al. (2014)
	Eckol, dieckol, PFF-A	MRC-5 human fetal lung fibroblast, RAW264.7 mouse macrophage, HL-60 human promyeloblast cell lines	Li et al. (2009)
AAPH	Eckol, dieckol	Zebrafish embryos	Kang et al. (2013)
High glucose	Dieckol	Liver and muscle tissue from C57BL/KsJ-*db*/*db* mice (model of type 2 diabetes)	Kang et al. (2013)
	Dieckol	INS-1 rat pancreatic β-cell insulinoma cell line	Lee et al. (2012)
High-fat diet	EEC	Kidney tissue from C57BL/6 mouse	Eo et al. (2017)
	EEC	Liver tissue from C57BL/6 mouse	Eo et al. (2015)

Phlorotannins reduce intracellular ROS levels and increase cell viability after the induction of oxidative stress by H_2_O_2_, AAPH, and a high-glucose and high-fat diet. PFF-A, phlorofucofuroeckol A; EEC, ethanol extract of *Ecklonia cava*; AAPH, 2,2′-azobis(2-amidinopropane) dihydrochloride.

## 5. Effects of phlorotannins on stroke

Stroke is the second leading cause of death globally, with 12 million new cases recorded every year (Feigin et al., 2022). In stroke, the blockage or rupture of cerebral arteries leads to ATP depletion, excitotoxicity and oxidative stress, resulting in apoptosis and edema (Lo et al., 2003; Chen et al., 2021). Treatment with a phlorotannin-rich extract of *E. cava* had protective effects against transient focal ischemia in rat brains (Kim et al., 2012). Treatment before and after the surgical occlusion of the middle cerebral artery decreased infarct size and edema in the brain in a dose-dependent manner (Kim et al., 2012). The number of apoptotic cells in the cerebral cortex and striatum was also significantly reduced, and higher neurological scores were observed at 2 to 6 days post-infarct.

## 6. Effects of phlorotannins on sleep

Insufficient or poor quality of sleep negatively affects cognitive functions such as reasoning, memory and learning (Stickgold, 2005; Alhola and Polo-Kantola, 2007). In a pilot study, human subjects who took a phlorotannin supplement experienced reductions in wakefulness after sleep onset as well as total wake time (Um et al., 2018). In ICR rats, treatment with *E. cava* extracts led to a dose-dependent increase in the number of animals that fell asleep when co-administered with a sub-hypnotic dose of pentobarbital (Cho et al., 2012). Co-administration of *E. cava* extract with sub-hypnotic (Cho et al., 2012) or hypnotic (Cho et al., 2012, 2014) doses of pentobarbital also led to increased sleep duration and decreased sleep latency in ICR rats. In mice, electroencephalogram and electromyogram studies revealed that treatment with an *E. cava* extract (Cho et al., 2014; Yoon et al., 2014) or dieckol (Yoon et al., 2020) accelerated the onset of sleep and increased the proportion of non-REMS, without affecting REMS. The effects of dieckol were comparable to those of zolpidem, a sedative and hypnotic drug (Yoon et al., 2020).

Flumazenil, a GABA_A_-benzodiazepine receptor antagonist, completely abolished the sleep-promoting effects of phlorotannins (Cho et al., 2012, 2014; Yoon et al., 2020). As dieckol, eckol, and other phlorotannins were shown to bind competitively against flumazenil to the GABA_A_-benzodiazepine receptor in a cell-free assay (Cho et al., 2012), these compounds are hypothesized to induce sedation by promoting GABAergic pathway activity.

## 7. Effects of phlorotannins on nociception

Pretreatment with dieckol was shown to exert analgesic effects in a study of Swiss albino mice (Li et al., 2021). After dieckol administration in drinking water, the responses to somatic, visceral, and neuropathic pain were tested using various methods such as the hot plate test, tail immersion in hot water, injection of pain-inducing agents into the paw, and intraperitoneal injection of acetic acid. Dieckol reduced pain responses to all tested stimuli in a dose-dependent manner, and these analgesic effects were observed at 30 to 120 min after treatment.

Interestingly, dieckol-treated mice showed significantly lower locomotive activity in an open field test in the absence of external stimuli, with reductions of up to 20% in the number of squares walked. A similar effect was observed in mice treated with 5 mg/kg morphine as a positive control. This decrease in locomotion may be associated with the sedative effects of phlorotannins, and/or the suppression of apomorphine-induced rotation after phloroglucinol treatment in a mouse model of PD (Ryu et al., 2013).

## 8. Pharmacological use of phlorotannins

Phlorotannins have many advantages as potential therapeutic candidates for CNS diseases. A pharmaceutically standardized phlorotannin-rich formulation (PH100) has been approved as an investigational new drug by the United States Food and Drug Administration, and several clinical trials have reported the absence of adverse effects in humans after supplementation with *E. cava* extracts (Shin et al., 2012; Choi et al., 2015; Lee and Jeon, 2015). Additionally, the European Food Safety Authority has classified *E. cava* phlorotannins as safe for consumption by healthy individuals (N. Efsa Panel on Dietetic Products et al., 2017). Recent phase II clinical trials have yielded promising results as potential treatments for metabolic diseases such as hypercholesterolemia and hyperglycemia (Lee et al., 2012; Shin et al., 2012; Choi et al., 2015; Lee and Jeon, 2015; Lee et al., 2018). The efficacy of phlorotannins as therapeutic agents for neurodegenerative diseases remains to be tested in humans.

## 9. Future directions

Many molecular mechanisms of action of phlorotannins have been proposed. *In vitro*, colorimetric assays have shown that dieckol, eckol, and PFF-A inhibit the activity of AChE (Myung et al., 2005; Yoon et al., 2008; Kannan et al., 2013; Choi et al., 2015), BuChE (Choi et al., 2015; Nho et al., 2020), MAOs (Jung et al., 2017; Seong et al., 2019), and GSK-3β (Choi et al., 2015). Phlorotannins have also been shown to regulate the biosynthesis (Jung et al., 2010; Kang et al., 2013; Choi et al., 2015) and oligomerization (Kang et al., 2011; Seong et al., 2019) of Aβ, as well as the neuroinflammatory (Lee et al., 2018; Yang et al., 2021) and neurotoxic (Kang et al., 2011; Ahn et al., 2012; Yang et al., 2015) pathways related to Aβ (Figure 3). In animal experiments, phlorotannins exerted neuroprotective effects against the development and progression of AD (Yang et al., 2015, 2018), PD (Ryu et al., 2013; Figure 4), and other CNS disorders (Cho et al., 2012; Kim et al., 2012), and yielded measurable improvements in behavioral aspects such as learning (Lee and Stein, 2004), memory (Myung et al., 2005), movement (Li et al., 2021), sleep (Cho et al., 2012, 2014; Yoon et al., 2014; Yoon et al., 2020), and symptomatic pain (Li et al., 2021; Table 1). The anti-inflammatory (Table 2) and antioxidative effects (Table 3) of phlorotannins may provide generally neuroprotective effects in the brain. However, observations from *in vitro* and *in vivo* experiments must be pieced together by demonstrating a causal relationship between the observed molecular changes and behavioral changes in animal models, in order to establish phlorotannins as effective drug candidates for the treatment of CNS disorders. For example, although phlorotannins disrupt the biosynthesis, aggregation, and neurotoxicity of Aβ species, it is unknown whether dieckol, eckol, and PFF-A can improve behavior and cognitive function in 5XFAD AD mice. Another example is in PD research, where changes in dopamine metabolism or mitochondrial function have not been correlated with behavioral and cognitive changes in animal models of PD.

In particular, a mechanistic understanding of phlorotannins at the cellular and circuit levels is lacking. To address this gap, the synaptic functions of dopaminergic, cholinergic, and GABAergic neurons and their interconnectivity can be examined in various brain circuits that direct cognition and behavior. In animal models of disease, electrophysiological, optical imaging, and optogenetic measurements after phlorotannin treatment can reveal changes in channel and receptor activity, membrane properties, temporal discharge pattern, or synaptic transmission and plasticity. Such studies would reveal how phlorotannins rescue behavioral symptoms and help to identify clear neuropharmacological targets of these compounds. For example, how phloroglucinol prevents the 6-OHDA-induced loss of DA neurons while suppressing the overactivation of DA receptors (apomorphine-induced rotation behavior) can be explored through electrophysiological measurement. Other questions to be addressed include how phlorotannins affect nociception, which involves multiple layers of neural circuits, or modulate GABA signaling to promote sleep. A systematic and thorough investigation of the effects of phlorotannin compounds, particularly using various electrophysiological technologies, is urgently needed.

## 10. Perspective

Phlorotannins may regulate neuronal excitability by modulating ion channels and receptors, such as those affecting K^+^ and Ca^2+^ homeostsis. Hyperexcitability can be a driver of various brain disorders including AD (H. Targa Dias Anastacio et al., 2022) and PD (Lawson, 2000), and low threshold voltage-gated A-type transient potassium (I_A_) channels are known to affect the excitability of neurons (Chien et al., 2007; Yang et al., 2015; Tetteh et al., 2018; Noh et al., 2019). In AD patients, the hippocampus and cortex display hyperexcitability (Dickerson et al., 2005; Olazaran et al., 2010), and Aβ species may block I_A_ channels to increase Ca^2+^ influx and cause dendritic hyperexcitability (Xu et al., 1998; Chen, 2005). With regards to PD, several features of DA neurons in the SNpc increase their susceptibility to neuronal death and contribute to parkinsonism. For example, subthreshold Ca^2+^ currents drive autonomous, pacemaker-like firing in the SNpc (Chan et al., 2007; Guzman et al., 2009), resulting in large Ca^2+^ oscillations and neuronal death. In the neighboring ventral tegmental area, pacemaking is driven by Na^+^ rather than Ca^2+^ currents, and less neuronal death occurs in this brain region (Khaliq and Bean, 2010). Glutamatergic activation of NMDARs also initiates burst firing and triggers an influx of Ca^2+^, further contributing to calcium-mediated excitotoxicity (Wang et al., 2020). In addition, SNpc DA neurons have comparatively lower cytoplasmic Ca^2+^ sequestering capacity due to lower calbindin levels (Gibb, 1991) and lower mitochondrial mass (Verkhratsky, 2002). High levels of cytosolic Ca^2+^ in SNpc DA neurons may cause neuronal death via apoptosis or necrosis (Blandini et al., 1996) via the activation of nitric acid synthase and phospholipase A2, which produce ROS (Blandini et al., 1996; Wang et al., 2020). Excess Ca^2+^ may also affect the release of neurotransmitters into the synapse.

**Figure 5:**
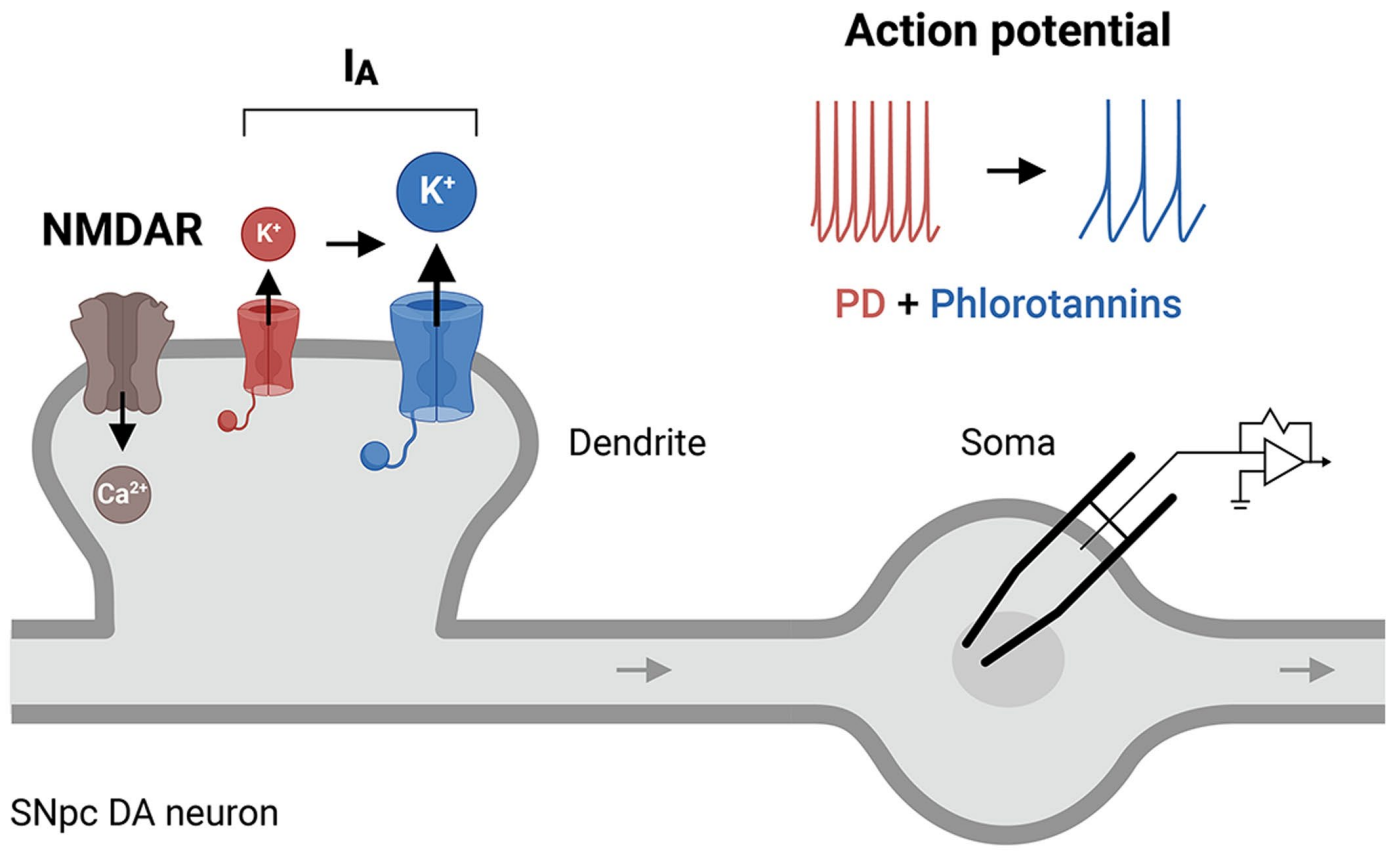
A putative cellular mechanism of phlorotannins to restore I_A_ receptor activity. Phlorotannin treatment may upregulate I_A_ receptors on the presynaptic membrane, thus altering the NMDAR/I_A_ ratio and dendritic excitability, and decreasing the frequency of action potential firing in SNpc DA neurons. These changes may reduce intracellular Ca^2+^ levels and thus counteract the excitotoxicity and pathogenesis of PD. PD, Parkinson’s disease; NMDAR, N-methyl-D-aspartate receptor; SNpc, substantia nigra pars compacta; DA, dopamine.

In dopaminergic neurons, dendritic excitability is largely determined by NMDARs (a Ca^2+^-permeating channel (Jones and Gibb, 2005)), I_A_ channels, or their interactions (Noh et al., 2019). I_A_ channels fine-tune the frequency of the Ca^2+^-dependent pacemaker such that downregulation or dysfunction of these channels causes an increase in the intrinsic firing rate (Liss et al., 2001; Amendola et al., 2012), leading to excessive Ca^2+^ loading and excitotoxicity. I_A_ channels have been implicated in the degeneration of the SNpc (Sarkar et al., 2020) and are abnormally expressed in the putamen of patients with PD (Noh et al., 2019). Dysregulation of I_A_ channels in the SNpc can shift the neuronal excitation/inhibition balance (e.g., the NMDAR/I_A_ ratio), leading to neuronal hyperexcitability and an increase in cytoplasmic Ca^2+^ (Lawson, 2000), which underlie the pathogenesis of PD. We question whether phlorotannins might regulate the activity of I_A_ channels directly and/or indirectly (Figure 5), given the strong involvement of I_A_ channel activity in CNS diseases (Noh et al., 2019) and the finding that phloroglucinol treatment improved motor performance in a mouse model of PD (Ryu et al., 2013). Oxidative impairment of Kv4.3 channels also selectively increases the firing frequencies of nigral dopaminergic neurons (Subramaniam et al., 2014). This increase may dysregulate intracellular Ca^2+^ levels and ultimately induce the onset of PD. A phlorotannin-mediated therapeutic mechanism involving I_A_ channels has been little studied in animal models of AD or PD, and further research is required to delineate the precise contribution of I_A_ channels to various neurodegenerative diseases.

## Author contributions

YJK, H-CS, and SY designed the study, conducted the investigation, and wrote the paper. YJK created figures. YJK, OIK, HJH, H-CS, and SY proofread the paper. SY acquired funding. All authors contributed to the article and approved the submitted version.

## Funding

This work was supported by the Research Grants Council of Hong Kong (11102618 and 11101922) for SY.

## Conflict of interest

YJK was previously employed, and OIK is currently employed by Botamedi Brain Health and Medical Care Company Limited.

The remaining authors declare that the research was conducted in the absence of any commercial or financial relationships that could be construed as a potential conflict of interest.

## Publisher’s note

All claims expressed in this article are solely those of the authors and do not necessarily represent those of their affiliated organizations, or those of the publisher, the editors and the reviewers. Any product that may be evaluated in this article, or claim that may be made by its manufacturer, is not guaranteed or endorsed by the publisher.
